# Activities of psychiatrists in specialized coronavirus disease 2019 wards at Juntendo Hospital

**DOI:** 10.1002/pcn5.228

**Published:** 2024-07-25

**Authors:** Yoshihide Takeshita, Narimasa Katsuta

**Affiliations:** ^1^ Department of Psychiatry Juntendo University Faculty of Medicine Tokyo Japan; ^2^ Division of Medical Education Juntendo University Faculty of Medicine Tokyo Japan; ^3^ Department of Safety and Health Promotion Juntendo University Tokyo Japan

**Keywords:** COVID‐19, pandemic, psychiatric care, psychiatric symptom, psychiatrist

## Abstract

Since the onset of the coronavirus disease 2019 (COVID‐19) pandemic in 2020, specialized COVID‐19 wards have been established in general hospitals across Japan. Juntendo Hospital also established a dedicated COVID‐19 ward; however, many hospitalized patients were found to have psychiatric symptoms, such as delirium and depression. Juntendo Hospital's COVID‐19 specialist beds were staffed mainly by internists, who specialized in physical illnesses and were unfamiliar with psychiatric symptoms, making it difficult for them to provide adequate treatment. Some staff members were also found to be suffering from mental illness, compounding these issues. In 2021, to address these challenges, Juntendo Hospital's psychiatry department began having psychiatrists make rounds once a week in specialized COVID‐19 wards. The number of consultations varied depending on the status of the COVID‐19 epidemic; however, in the peak month, 45 consultations were made per month. Most consultations involved delirium and neurotic conditions, and there had been over 200 consultations for both by August 2023. We addressed not only the mental symptoms of the patients, but also the health status of the staff at the hospital beds, and took measures to maintain the mental health of the staff. Consequently, the hospital has not experienced any large‐scale medical breakdowns due to excessive staff fatigue. New pandemics of emerging infectious diseases will likely occur in the future, and we believe that we need to learn from this pandemic and prepare for future pandemics.

## Graphical abstract

Juntendo Hospital established a dedicated COVID‐19 ward in 2020, but many patients were found to have psychiatric symptoms such as delirium and depression. For this reason, starting in 2021, Juntendo Hospital's psychiatry department began having psychiatrists make rounds once a week in specialized COVID‐19 wards. As a result, our hospital has not experienced any large‐scale medical breakdowns due to excessive staff fatigue.

## INTRODUCTION

A new type of viral pneumonia was discovered in Wuhan, China, by the end of 2019. This disease, caused by severe acute respiratory syndrome coronavirus 2 (SARS‐CoV‐2) and named coronavirus disease 2019 (COVID‐19), evolved into a pandemic. The first case in Japan was confirmed in January 2020. Subsequently, the Ministry of Foreign Affairs issued a recommendation at the end of March 2020^1^ to suspend non‐essential overseas travel. However, despite the government response, the number of infected people quickly increased, and a state of emergency was declared in April 2020.

COVID‐19 is characterized by a long incubation period and strong infectivity as compared with that of conventional viral infections such as influenza. Initially, no treatment was established. The symptoms in young individuals are mild, comprising mainly fever, cough, and a loss of taste and smell. However, in the older population, the disease can lead to fatal pneumonia and respiratory failure, contributing to an increased number of severe cases. The number of people experiencing depression and anxiety also increased with the declaration of a state of emergency and the implementation of lockdowns in overseas cities to prevent infection.^2^ Many hospitalized patients also reportedly exhibited psychiatric symptoms because of the hospital environment.^3^ At the beginning of the epidemic, there were fears of infection and unfair discrimination against medical workers, leading to severe exhaustion among medical workers globally.^4^ Burnout was a prevalent issue as well.^5^

Given the highly contagious nature of the disease, the risk of in‐hospital infection surged, and none of the hospitals could handle the situation with existing beds, therefore they opened new wards specializing in infectious diseases. In Japan, Juntendo Hospital opened a specialized COVID‐19 ward in March 2020, and each clinical department worked together to respond to the pandemic.

Psychiatrists are in high demand in specialized COVID‐19 wards to manage patients with psychiatric symptoms and maintain the mental health of the staff. In Japan, many hospitals have created specialized liaison teams,^6^ and the role of psychiatrists at general hospitals has been brought into close focus.

## COVID‐19 TREATMENT AT JUNTENDO HOSPITAL

Juntendo Hospital, located in the center of Tokyo, serves as both a university hospital and a special function hospital. There are approximately 80 special function hospitals across Japan. According to Juntendo Hospital's published data,^7^ the number of annual consultations in 2021 was 1500 for first visits and 32,000 for repeat visits. The hospital was extremely busy, with an average of over 900 hospitalized patients managed daily.

Juntendo Hospital began admitting patients with COVID‐19 in March 2020. By August 2020, beds for patients with severe or moderate symptoms and those with mild symptoms were opened. In the former case, the open space in the intensive care unit was renovated to include private rooms. Although the number of beds varies depending on the season, there are approximately 25 beds for mildly ill patients and 15 for severely ill patients. One floor of the general hospital was used for the latter. In creating specialized wards, we isolated contagious patients from the conductors as much as possible by having the patients and staff use separate entrances and exits. Other considerations were made to prevent nosocomial infections, such as establishing the nursing station as a clean zone and wearing protective clothing in and near the patients' rooms.

Since April 2020, the COVID‐19 treatment team has been led by doctors from the general medicine and emergency departments, with support from internal medicine doctors, including those from the respiratory medicine department.

## PSYCHIATRISTS IN COVID‐19 TREATMENT

The Psychiatry Department of Juntendo Hospital has approximately 15 full‐time doctors. On average, a full‐time doctor sees approximately 20 patients during half‐day outpatient visits twice a week. A total of 15 beds for hospitalization were in closed wards. Although there are only 15 wards, many psychiatric patients with severe physical illnesses were hospitalized and underwent modified electroconvulsive therapy. The first author was in charge of the ward and was responsible for treating inpatients and managing the hospital ward while performing outpatient work.

When the physical medicine medical treatment team was established in April 2020, we launched the COVID‐19 mental care team (hereafter referred to as the mental care team) at the request of the hospital. However, when launched in 2020, it could not contribute to the COVID‐19 medical team because of the problems described below. We were deeply concerned regarding this situation because we understood the need for psychiatrists in COVID‐19 care, therefore, since 2021, the mental care team has made major changes to the medical care system in the hospital.

The targets of the mental care team were hospitalized patients and the staff who attended to them. A physician in charge of on‐campus treatment within the Department of Psychiatry handled the staff. The patients were treated by a liaison doctor, who changed the treatment schedule daily. We have established a system that allows 24‐h support from a psychiatrist on duty, even on holidays. Initially, our policy was to wait until we received a request from a doctor treating patients with COVID‐19. However, in 2020, almost no requests were received. At that time, patients with COVID‐19 were legally required to stay in isolation facilities for approximately 10 days, and many were discharged from the hospital within a relatively short period, therefore, in many situations, patients were promptly discharged from the hospital, even if they developed psychiatric symptoms. This practice was different when the psychiatric symptoms were severe. However, in cases where the symptoms were relatively mild, opportunities for psychiatric consultation were often missed.

Two other issues were also problematic at that time.

a) Staff care
  Medical staff worldwide tended to be depressed from the early stages of the COVID‐19 epidemic.^8^ At our hospital, staff became exhausted as the pandemic spread. The results of the Center for Epidemiologic Studies Depression Scale administered to Juntendo Hospital staff in 2020^9^ showed that the tendency for depression increased significantly compared with that of the previous year, especially among nurses with previously low depression levels. Some of these staff members wished to see a psychiatrist. Previous overseas studies have also pointed out that many nurses who cared for patients with COVID‐19 exhibit depression and anxiety.^10^ However, our hospital's policy for psychiatric examinations was to come directly to the hospital, which meant that busy staff members could not attend. In addition, the scope of response was limited to individual treatment, and there were no measures from a public health perspective, such as maintaining the mental health of the entire department or preventing deterioration. Therefore, the issue of not having a place where all staff could seek counseling to help stabilize their mental state has often been raised at hospital meetings.
b) Increase in the number of hospitalized patients
  Compared with during April 2020, the domestic polymerase chain reaction (PCR) testing system for COVID‐19 expanded around the fall of the same year. According to data from the Ministry of Health, Labour and Welfare,^11^ the number of PCR tests conducted daily from April to July 2020 was 10,000. The testing system was improved in September 2020, and by the end of the same year, 30,000 PCR tests were conducted daily.
  As a result, more patients who had been overlooked were identified, and the number of hospitalized patients increased. In addition, viral mutations increased infectivity. Furthermore, as society became exhausted by the prolonged pandemic and infection control measures, the number of infected people increased dramatically by the end of 2020. From April to July 2020, the maximum number of infected individuals per day in Japan was 663; on most days, it was fewer than 100. The number of infected people increased after November 17, 2020; it was 4524 on December 31, 2020, and the number of infected people diagnosed per day was between 2000 and 3000.
c) Improvements after 2021
  The mental health care team has significantly changed its medical care system since the beginning of 2021 to address these issues. A full‐time psychiatrist conducted rounds in both wards every Friday morning. At this time, a conference was held with the ward staff, and appropriate interventions were conducted for the patients as deemed necessary. We also regularly met with management staff in both wards to discuss the mental health of the ward staff. A psychologist within the Psychiatry Department was consulted, and an industrial physician at the university then lectured the staff on maintaining mental health. The patients regularly participated in meetings related to in‐hospital COVID‐19 treatment. Through this, information was shared with the hospital's operational department to optimize ward management. Consequently, the number of patient‐related consultations increased. In addition, by negotiating directly with the staff during their rounds, they began to receive a wide range of consultations from medical professionals such as doctors and nurses.

## DIFFERENCES BETWEEN REGULAR LIAISON CARE AND LIAISON CARE IN SPECIALIZED COVID‐19 WARDS

COVID‐19 is more contagious than conventional seasonal influenza^12^, ^13^, ^14^, ^15^ and is mainly transmitted by droplets after an incubation period of several days. In particular, it is likely to become severe in patients with a history of respiratory or cardiovascular disease or with immunosuppressive states such as those caused by oral steroids. Consequently, the number of seriously ill patients increases if the number of patients with a history of respiratory or cardiovascular disease is high. Furthermore, it is easy for mass infections to occur in hospitals and nursing homes, especially in older people, given the high infectivity of SARS‐CoV‐2. Overseas, there have been reports that the excess mortality rate for patients in institutional settings at the beginning of the pandemic was significantly higher than that for patients living at home.^16^ For this reason, specialized COVID‐19 wards require more thorough infection protection than regular liaison care. Being unable to visit their families and required to be confined to their rooms,^17^ patients often developed delirium^18^, ^19^ due to a reversal of their day/night rhythm owing to a decrease in physical activity and external stimulation. Furthermore, medical staff were required to wear protective equipment at all times and minimize face‐to‐face consultations to reduce the risk of horizontal transmission (especially at the beginning of the pandemic). Consequently, opportunities for non‐verbal communication, such as checking facial expressions on both sides, decreased, and the stress of the patients increased. The increased mortality rate and absence of established treatment at the beginning of the pandemic also contributed to severe anxiety in the patients. Several patients complaining of depression and insomnia^20^ as psychogenic reactions to this situation were also a characteristic of liaison care in COVID‐19 beds.

## TRENDS IN THE NUMBER OF CONSULTATIONS

**Figure 1:**
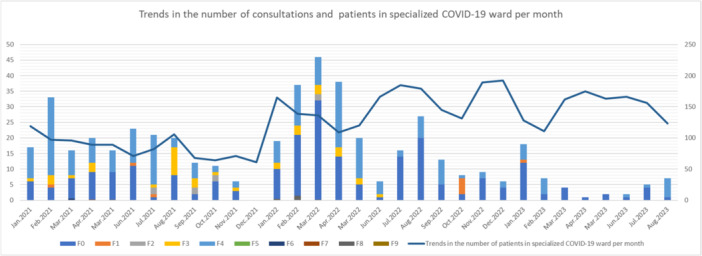
Trends in the number of consultations per month. The *x* axis represents the number of consultations. The bar graph shows the number of consultations per month color‐coded by disease. The line graph shows trends in the number of patients with COVID‐19 admitted to Juntendo Hospital. The *y* axis represents the month. F0: Organic, including symptomatic, mental disorders, F1: Mental and behavioural disorders due to psychoactive substance use, F2: Schizophrenia, schizotypal and delusional disorders, F3: Mood [affective] disorders, F4: Neurotic, stress‐related and somatoform disorders, F5: Behavioural syndromes associated with physiological disturbances and physical factors, F6: Disorders of adult personality and behaviour, F7: Mental retardation, F8: Disorders of psychological development, F9: Behavioural and emotional disorders with onset usually occurring in childhood and adolescence.

As mentioned earlier, the psychiatrist began regular ward rounds in January 2021. Immediately after the clinical rounds began, we received inquiries regarding the patients' mental symptoms. For cases brought up in conferences with ward staff, we checked medical records, adjusted prescriptions, and conducted direct medical examinations as necessary. We performed remote examinations in the hospital using simple mobile phones and tablet devices to prevent nosocomial infections. The patients were directly examined while wearing an N95 mask and other protective equipment, as this type of examination is impossible in a hospital ward for severely ill patients. The number of consultations changed significantly from month to month because of an increased number of hospitalized patients due to the spread of mutant strains. The consultation trends up to the middle of 2023 are summarized in Figure 1. The line graph shows the number of patients with COVID‐19 admitted to Juntendo Hospital. Until the summer of 2021, there were many patients diagnosed with F4; however, from the beginning of 2022, the number of patients diagnosed with F0 increased. The Ethics Committee of Juntendo Hospital approved the publication of these data.

According to statistics from the Digital Agency,^21^ the percentage of individuals fully vaccinated against COVID‐19 reached 70% by the end of October 2021. The number of infected people in Japan decreased as the vaccination rate increased^22^; the number of consultations significantly decreased between November and December 2021. The so‐called ο strain appeared between January and March 2022. According to the data above from the Ministry of Health, Labour and Welfare, the total number of infected people in Japan at the time was at a record high every day, with single‐day cases exceeding 100,000 people on some days. In some months during the same period, the number of monthly consultations exceeded 40. The number of consultations began to decline after February 2023, when the proportion of severely ill people among the infected population decreased. In May 2023, COVID‐19 was downgraded to a level equivalent to that of Category 5 under the Infectious Disease Control Law, and the current level continues to be low.

## DISEASE BREAKDOWN

**Figure 2:**
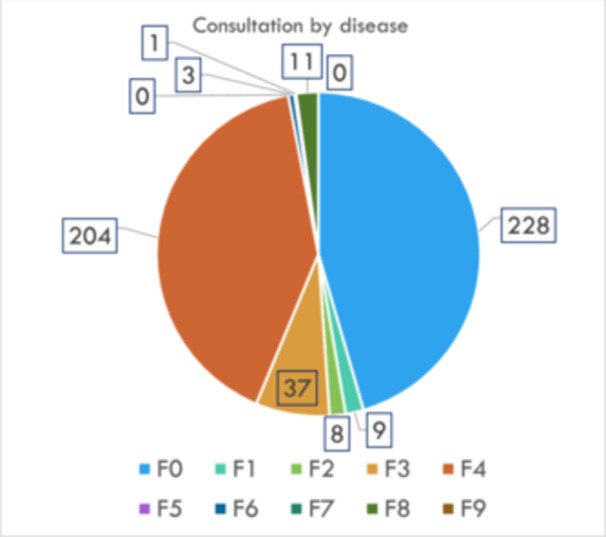
Consultation by disease. The pie chart shows the number of consultations by disease (expressed using International Classification of Diseases, 10th revision, codes).

As discussed above, there was more than one consultation per day, but the trends shown in Figure 2 were observed when broken down by disease. This figure shows the consultation topics categorized by disease from January 2021 (i.e., when we started working in the specialized COVID‐19 ward) to August 2023.

The International Classification of Diseases (10th revision) codes are listed in the figure. The most common cases were those classified as organic mental disorders, such as dementia and delirium. Regarding delirium, more than 25% of older patients with COVID‐19 who visit the emergency department reportedly have delirium.^23^ In such cases, medical treatment was seriously hampered by self‐removal of intravenous fluids and tubes and violent resistance to nursing care. This behavior is probably why these patients accounted for most consultations with psychiatrists. For these cases, we adjusted the medications that induced delirium and created a day/night rhythm using non‐benzodiazepine drugs. Simultaneously, the hospital environment improved orientation, and the patient's symptoms improved.

Many neurotic disorders classified as F4, such as adjustment disorders, have been reported previously. In Japan, where the number of people with COVID‐19 was lower than that in Western countries, there was considerable stigma surrounding the infected people. According to the group work of the Subcommittee on Novel Coronavirus Disease Control,^24^ sponsored by the Japanese government's Cabinet Secretariat, infected people were fired from their workplaces and their personal information was maliciously posted on social networking services. There have been reports of incidents such as privacy violations. Even after recovering from the infection, some infected people are socially criticized and isolated, and the infected people treated at our hospital often blame themselves for their infection. In these cases, direct examinations were conducted using protective equipment to alleviate anxiety and depression. In addition, symptoms improved in many of these patients after combining anxiolytic drugs and sleeping pills.

Among cases initially classified as F4, symptoms of mood disorders such as depression were occasionally observed because of prolonged symptoms and prolonged hospitalization. In rare cases, patients developed withdrawal delirium because of a lack of alcohol intake in a hospital environment, whereas others developed a detention reaction in a closed environment.

## IMPORTANCE OF FACE‐TO‐FACE COMMUNICATION

Regular rounds once a week improved communication with ward staff, such as doctors and nurses. In our hospital, the general medical department, which is not specialized in psychiatry, was converted into a specialized COVID‐19 ward. At the beginning of 2021, there were voices of confusion about how to deal with patients with psychiatric symptoms. In particular, many nurses and medical practitioners expressed concerns regarding assessing the symptoms of low‐activity delirium and providing explanations to patients with mental illness. Improving the hospital environment that causes delirium and using medications as needed at the appropriate time have become possible by approaching these issues from an educational perspective. Physicians also asked many questions regarding the use of internal medicines that cause psychiatric symptoms and medications in restless patients at a high risk of aspiration. We actively discussed and shared knowledge regarding these issues with the physicians during the rounds.

In addition, by 2022, clusters occurred in multiple wards, resulting in staff absence from work. After the rounds, the psychiatrist visited these wards to check whether the staff felt excessive remorse. When necessary, interviews were conducted with psychologists and industrial physicians. When the infection was under control, we consulted the personnel in charge of each clinical department and ward supervisor, and conducted awareness campaigns to maintain mental health. Our hospital did not experience medical collapse due to excessive fatigue of the medical staff, as seen in hospitals in Western countries because of these activities.

## CONCLUSION

Since 2020, owing to the tremendous efforts of the staff at each hospital, the 3‐year COVID‐19 pandemic in Japan has ended. However, the COVID‐19 pandemic has brought to light racial discrimination and social divisions. In this respect, it is quite different from past infectious diseases and has left a huge scar on the society.^25^ Since World War II, Japan has not experienced any outbreaks of infectious diseases that could change its social structure and has been behind the curve in its response to such outbreaks. As those responsible for the activities of our hospital's Psychiatry Department, we cannot help but feel that “it's better to be rough and ready than slow and elaborate.”

The possibility of new pandemics of infectious diseases is increasing. In the future, it will be necessary to focus on the activities of psychiatrists on a global level during the previous pandemic to prepare for the next pandemic.

## AUTHOR CONTRIBUTIONS

Yoshihide Takeshita interviewed and treated the patient and wrote and revised the first draft of the manuscript. Narimasa Katsuta1 rewrote and revised the manuscript. All authors contributed to and approved the final version of the manuscript.

## CONFLICT OF INTEREST STATEMENT

The authors declare no conflict of interest.

## ETHICS APPROVAL STATEMENT

This study adhered to the “Ethical Guidelines for Medical Research for Humans” and the Declaration of Helsinki. The Juntendo University School of Medicine Ethics Committee (Approval No. H21‐0102) approved the research protocol.

## PATIENT CONSENT STATEMENT

Because this was a retrospective study that dealt with existing medical records, the requirement for informed consent was waived. Information about the study design was posted on the Juntendo University Hospital website, and all candidates were guaranteed the opportunity to refuse to participate in the study.

## CLINICAL TRIAL REGISTRATION

N/A.

## Data Availability

Research data are not shared. The raw data belonging to this study cannot be made publicly available because the disclosure of personal data was not included in the research protocol of this study. The data are not publicly available due to privacy and ethical restrictions.
